# A pre-post quasi-experimental study of antimicrobial stewardship exploring the impact of a multidisciplinary approach aimed at attaining an aggressive joint pharmacokinetic/pharmacodynamic target with ceftazidime/avibactam on treatment outcome of KPC-producing *Klebsiella pneumoniae* infections and on ceftazidime/avibactam resistance development

**DOI:** 10.1128/aac.00488-25

**Published:** 2025-06-06

**Authors:** Milo Gatti, Matteo Rinaldi, Pier Giorgio Cojutti, Cecilia Bonazzetti, Antonio Siniscalchi, Tommaso Tonetti, Simone Ambretti, Sara Tedeschi, Maddalena Giannella, Pierluigi Viale, Federico Pea

**Affiliations:** 1 Department of Medical and Surgical Sciences, Alma Mater Studiorum University of Bologna 198207, Bologna, Italy; 2 Clinical Pharmacology Unit, Department for Integrated Infectious Risk Management, IRCCS Azienda Ospedaliero-Universitaria di Bologna 18508 , Bologna, Italy; 3 Infectious Diseases Unit, Department for Integrated Infectious Risk Management, IRCCS Azienda Ospedaliero-Universitaria di Bologna 18508 , Bologna, Italy; 4 Anesthesia and Intensive Care Medicine, IRCCS Azienda Ospedaliero-Universitaria di Bologna 18508 , Bologna, Emilia-Romagna, Italy; 5 Division of Anesthesiology, Department of Anesthesia and Intensive Care, IRCCS Azienda Ospedaliero-Universitaria di Bologna 18508 , Bologna, Italy; 6 Operative Unit of Microbiology, Department for Integrated Infectious Risk Management, IRCCS Azienda Ospedaliero-Universitaria di Bologna 18508 , Bologna, Italy; Columbia University Irving Medical Center, New York, New York, USA

**Keywords:** ceftazidime/avibactam, KPC-producing *Klebsiella pneumoniae*, continuous infusion, TDM-guided approach, aggressive joint PK/PD target, multidisciplinary approach, resistance development, microbiological failure

## Abstract

To assess the impact of a multidisciplinary approach aimed at attaining aggressive joint pharmacokinetic/pharmacodynamic (PK/PD) target with ceftazidime/avibactam on treatment outcome of KPC-*Klebsiella pneumoniae* (Kp) infections and prevention of ceftazidime/avibactam resistance development, a pre-post quasi-experimental study on adult patients with documented KPC-Kp who were treated with ceftazidime/avibactam according to a multidisciplinary approach in the period 1 March 2021–31 October 2024 and patients receiving standard management with ceftazidime/avibactam in the period 1 January 2018–28 February 2021 was performed. Multivariate analysis was performed to identify variables associated with microbiological failure and 90-day resistance development to ceftazidime/avibactam in both pre- and post-intervention phases. A total of 116 and 102 patients in pre- and post-intervention phases were included. A significantly lower microbiological eradication rate (53.0% vs. 81.0%; *P* < 0.001), a lower clinical cure rate (48.3% vs. 70.6%; *P* < 0.001), and a higher rate of 90-day resistance development (15.5% vs. 5.9%; *P* = 0.02) were found in the pre-intervention phase. Continuous renal replacement therapy (odds ratio [OR] 5.20; 95% confidence interval [CI] 1.21–22.34) and a ceftazidime/avibactam MIC value ≥ 4 mg/L (OR 3.08; 95% CI 1.10–8.64) emerged as independent predictors of microbiological failure in the pre-intervention phase. Conversely, attaining aggressive joint PK/PD target (OR 0.03; 95% CI 0.005–0.20) and bloodstream infections (OR 0.09; 95% CI 0.02–0.53) resulted in protection against microbiological failure in the post-intervention phase. Attaining aggressive joint PK/PD targets resulted in protection against 90-day resistance development in the post-intervention phase (OR 0.07; 95% CI 0.01–0.69). Implementing a multidisciplinary approach for maximizing the attainment of aggressive joint PK/PD targets of ceftazidime/avibactam could represent an effective strategy for preventing resistance development to ceftazidime/avibactam in KPC-Kp infections.

## INTRODUCTION

The widespread diffusion of carbapenem-resistant *Enterobacterales* (CRE) is representing nowadays a big threat to global public health, accounting for remarkable morbidity, mortality, and healthcare costs (1, 2). Among CRE, the production of *Klebsiella pneumoniae* carbapenemase (KPC) is one of the most relevant underlying mechanisms, especially for *Klebsiella pneumoniae* (Kp) (2). Ceftazidime/avibactam is a beta-lactam/beta-lactamase inhibitor combination (BL/BLIc) currently recommended as first-line treatment of KPC-Kp-related infections by international guidelines and/or guidance (3, 4). Unfortunately, selective pressure associated with its ever-growing use has favored in the last years the appearance of KPC-Kp strains resistant to ceftazidime/avibactam, with prevalence rates as high as 15–20% in some complex settings (5–7). Consequently, adopting valuable strategies for counteracting this tendency in clinical practice is absolutely needed. Maximizing pharmacokinetic/pharmacodynamic (PK/PD) target attainment of ceftazidime/avibactam in patients could be worthwhile for this purpose, potentially becoming a mandatory issue for extending the lifetime of this agent.

Ceftazidime/avibactam is a time-dependent BL/BLIc for which PK/PD target attainment should deal with both the BL and the BLI, that is, should be joint. Recent findings suggested that translating into clinical practice the conservative joint PK/PD targets identified in preclinical models could not suffice for preventing resistance development to ceftazidime/avibactam (8, 9). In this regard, it was shown that targeting avibactam concentrations to higher thresholds could be helpful (10, 11) and that aiming at an aggressive joint PK/PD target attainment may represent the way forward for counteracting microbiological failure and resistance development in clinical practice (12–15). The use of prolonged infusion may increase the likelihood of attaining aggressive joint PK/PD targets under the same daily dose. A retrospective cohort study carried out among 577 patients having KPC-Kp infections being treated with ceftazidime/avibactam found that the use of prolonged infusion by improving PK/PD target attainment was associated with a significant decrease in the mortality risk compared to that of intermittent infusion (12). Unfortunately, it was recently shown that aggressive joint PK/PD target attainment may be hampered, especially in patients without renal dysfunction, by a much faster avibactam than ceftazidime elimination (16). This means that under these circumstances, the use of prolonged or even continuous infusion (CI) could not by itself warrant always adequate ceftazidime protection by avibactam against KPC-mediated hydrolysis. Consequently, implementing ceftazidime/avibactam delivery by CI coupled with a therapeutic drug monitoring (TDM)-guided approach should represent the best way to deal with these issues in clinical practice.

Based on these assumptions, this study would like to assess the impact of a multidisciplinary intervention of antimicrobial stewardship aimed at attaining aggressive joint PK/PD target with ceftazidime/avibactam on treatment outcome of KPC-Kp infections and prevention of ceftazidime/avibactam resistance development.

## MATERIALS AND METHODS

### Study design and inclusion criteria

This is a retrospective pre-post quasi-experimental study involving adult patients having documented KPC-Kp infections being treated with ceftazidime/avibactam in the period 1 January 2018–31 October 2024 at the IRCCS Azienda Ospedaliero-Universitaria of Bologna, Italy. Patients having only KPC-Kp colonization or being in compassionate care and dying within 48 h from starting ceftazidime/avibactam treatment were excluded. In the pre-intervention phase (from 1 January 2018, that is, the first date of ceftazidime/avibactam availability at our institution, to 28 February 2021), patients had treatment with ceftazidime/avibactam in mono or combination therapy based on infectious disease consultant advice according to the standard of care. In the post-intervention phase (from 1 March 2021 to 31 October 2024), patients had treatment with ceftazidime/avibactam based on a multidisciplinary intervention of antimicrobial stewardship agreed between the infectious disease consultants, the MD clinical pharmacologists, and the clinical microbiologists. This approach started with the establishment in March 2021 of a novel TDM-guided expert clinical pharmacological advice (ECPA) program of ceftazidime/avibactam. This innovation offered the opportunity to discuss together which steps would have been needed for maximizing ceftazidime/avibactam effectiveness and favoring as much as possible its use in monotherapy (17, 18). It was agreed that the infectious disease consultants would have been more confident in using ceftazidime/avibactam monotherapy by using CI administration (after loading) and by tailoring therapy through TDM-guided ECPAs aimed at aggressive joint PK/PD target attainment. The study was conducted according to the guidelines of the Declaration of Helsinki and approved by the local ethical committee (No. EM 232-2022_308/2021/Oss/AOUBo on 16 March 2022).

### Data collection and variable definition

Demographic (age, sex, and body mass index [BMI]), clinical/laboratory data [underlying diseases, admission ward, Charlson comorbidity index (CCI), presence of immunosuppression, baseline creatinine clearance (CL_CR_) estimated by means of the CKD-EPI formula (19), vasopressor use, need for mechanical ventilation and/or continuous renal replacement therapy (CRRT) and/or intermittent hemodialysis (IHD), occurrence of augmented renal clearance (ARC; defined as a normal serum creatinine level coupled with an estimated CL_CR_ > 130 mL/min/1.73 m^2^ in males and >120 mL/min/1.73 m^2^ in females) (20), microbiological data (site/type of infection, MIC values of the KPC-Kp strains), ceftazidime/avibactam treatment data (baseline daily dosing regimen, use in intermittent infusion (II) or CI, mono- or combination therapy, treatment duration, plasma ceftazidime and avibactam concentrations, aggressive joint PK/PD target attainment or non-attainment), and outcome data (microbiological eradication/failure, 90-day resistance development, clinical cure, 30-day mortality rate) were retrieved in both pre- and post-intervention phases.

Types of infection were defined according to the Centers for Disease Control and Prevention criteria (21). Specifically, bloodstream infection (BSI) was defined as the isolation of KPC-Kp from at least one blood culture (21). Hospital-acquired pneumonia (HAP) was defined as the isolation of KPC-Kp from the endotracheal aspirate culture with a bacterial load ≥ 10^6^ CFU/mL after more than 48 h of hospital admission (22). Ventilator-associated pneumonia (VAP) was defined as the isolation of KPC-Kp from the bronchoalveolar lavage fluid culture with a bacterial load ≥ 10^4^ CFU/mL after more than 48 h of endotracheal intubation and start of mechanical ventilation (22). Intra-abdominal infection (IAI) was defined as the isolation of KPC-Kp from the peritoneal fluid or from abdominal/biliary specimens (22). Urinary tract infection (UTI) was defined as the isolation of KPC-Kp from urine culture with a bacterial load ≥ 10^5^ CFU/mL (22). Skin and soft tissue infection (SSTI) was defined as the isolation of KPC-Kp from a biopsy sample of the advancing margin skin lesion (22). Bone and joint infection (BJI) was defined as the isolation of KPC-Kp from bone/tissue biopsy or from synovial fluid/biopsy (22).

Ceftazidime/avibactam susceptibility was tested by means of the broth microdilution method (panel provided by Merlin Diagnostika GmbH, Bornheim-Hersel, Germany). The tested MIC values of ceftazidime ranged from 0.5 to 64 mg/L in the presence of a fixed target avibactam concentration (*C*_*T*_) of 4 mg/L and were interpreted according to the European Committee on Antimicrobial Susceptibility Testing (EUCAST) guidelines (23). Ceftazidime/avibactam resistance was defined as an MIC value > 8 mg/L.

Microbiological eradication or failure was defined as the eradication from or the persistence at the infection site of the index KPC-Kp strain as documented by follow-up cultures after more than 7 days from starting ceftazidime/avibactam treatment (24). Resistance to ceftazidime/avibactam was defined as an MIC increase beyond the EUCAST clinical breakpoint of susceptibility of the index KPC-Kp strain or of any other clinical isolate yielded from a rectal swab within 90 days (23, 24). Clinical cure was defined as complete resolution of signs and symptoms of infection coupled with documented microbiological eradication at the end of treatment, absence of recurrence/relapse at 30-day follow-up, and attributable mortality due to KPC-Kp infection (25).

### Ceftazidime/avibactam treatment features

Ceftazidime/avibactam was used as first-line treatment of documented KPC-Kp infections in mono or combination therapy and delivered by II or CI at the discretion of the treating physician. Combination therapy was defined as the concomitant use with other antimicrobial agents active against KPC-Kp for at least 48 h.

Ceftazidime/avibactam treatment was always started with a loading dose (LD) of 2 g/0.5 g over 2 h, immediately followed by a maintenance dose (MD) selected on the basis of the patient’s estimate of CL_CR_ (2 g/0.5 g every 8 h if CL_CR_ > 50 mL/min/1.73 m^2^; 1 g/0.25 g every 8 h if CL_CR_ 31–50 mL/min/1.73 m^2^ or in case of CRRT; 0.75/0.185 g every 12 h if CL_CR_ 16–30 mL/min/1.73 m^2^; 0.75 g/0.185 g every 24 h if CL_CR_ 6–15 mL/min/1.73 m^2^; and 0.75 g/0.185 g every 48 h if IHD). Whenever using CI, stability of ceftazidime/avibactam was granted by reconstituting the aqueous solutions every 8 h maximum and by administering over 8 h (26).

In the pre-intervention phase, dosing adjustments during treatment were provided only whenever the patient had fluctuations in renal function. In the post-intervention phase, dosing adjustments were guided by a TDM-based ECPA aimed at attaining the aggressive joint PK/PD target of ceftazidime/avibactam, as described previously (14). Briefly, the aggressive joint PK/PD target was defined as the simultaneous attainment of a ceftazidime *fC*_ss_ or *fC*_min_/MIC ratio > 4 (equivalent to 100% *fT*_>4× MIC_) coupled with an avibactam *f*C_ss_ or *C*_min_/target concentration (*C*_T_) ratio > 1 (where *C*_T_ is the fixed target avibactam concentration threshold used by the EUCAST for testing the *in vitro* standard susceptibility of ceftazidime/avibactam, namely, 4 mg/L). Aggressive joint PK/PD target non-attainment was defined as the attainment of only one or none of the two thresholds (14). The first TDM-guided ECPA was performed after at least 24 h from starting therapy and reassessed every 48–72 h whenever feasible. Total ceftazidime and avibactam steady-state (in case of CI) or trough (in case of II) plasma concentrations (*C*_ss_ or *C*_min_) were determined by means of a validated liquid chromatography-tandem mass spectrometry method (27). The free fractions (*f*) of ceftazidime and avibactam were calculated by multiplying the total *C*_min_ or *C*_ss_ by 0.90 and 0.93 based on a plasma protein binding of 10 and 7%, respectively (28).

### Statistical analysis

Demographics and clinical characteristics of patients were summarized by using absolute frequencies and percentages for categorical variables and median with interquartile ranges (IQR) for continuous variables. Univariate analysis for testing potential differences between patients included in the two phases was performed by means of the Fisher’s exact test or the chi-squared test (for categorical variables), or the Mann–Whitney U test (for continuous variables).

Multivariate logistic regression analysis was performed for testing in each of the two intervention phase variables potentially associated with microbiological failure and/or with 90-day resistance occurrence to ceftazidime/avibactam. To minimize the risk of confounding factors, the model was adjusted for age and sex. All the independent covariates being associated with a *P* value < 0.10 at the univariate analysis were included in the multivariate logistic regression model. Statistical significance was defined as a *P* value < 0.05.

Statistical analysis was performed by means of MedCalc for Windows (MedCalc statistical software, version 19.6.1, MedCalc Software Ltd., Ostend, Belgium).

## RESULTS

**Fig 1:**
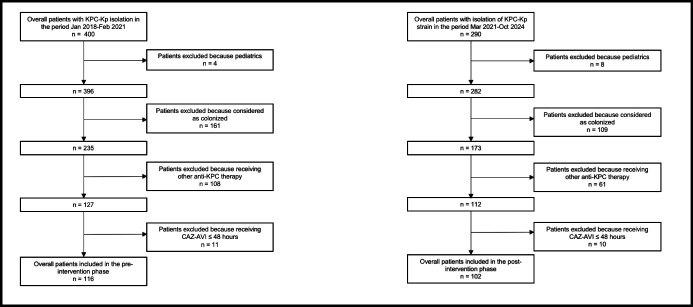
Flowchart of patients’ inclusion and exclusion criteria.

In the overall study period, a total of 690 patients had a microbiological culture positive for KPC-Kp (400 in the pre-intervention phase and 290 in the post-intervention phase). Among these, 218 were considered eligible for this study (116 in the pre-intervention phase and 102 in the post-intervention phase; Fig. 1). Demographics and clinical features are summarized in Table 1.

**TABLE 1:** Comparison of demographics and clinical characteristics of patients receiving ceftazidime-avibactam therapy for the management of KPC-producing *Klebsiella pneumoniae* infections between pre- and post-intervention^*b*^^,^^*c*^

Variables	Overall (*n* = 218)	Pre-intervention phase (*n* = 116)	Post-intervention phase (*n* = 102)	*P* value
*Demographics*				
Age (yrs; median; [IQR])	65.5 (56.3–74.0)	64.5 (53.0–74.0)	67.0 (60.0–73.8)	0.19
Gender (male/female; *n* [%])	145/73 (66.5/33.5)	82/34 (70.7/29.3)	63/39 (61.8/38.2)	0.16
Body mass index (kg/m^2^; median; [IQR])	25.3 (22.2–28.4)	25.1 (22.2–27.5)	25.4 (22.5–29.1)	0.25
Obesity	39 (17.9)	18 (15.5)	21 (20.6)	0.33
*Underlying disease* (*n*; [%])				
Solid organ transplant recipient	57 (26.1)	34 (29.3)	23 (22.5)	0.26
Solid cancer	31 (14.2)	19 (16.4)	12 (11.8)	0.33
Valvular/vascular prosthesis placement	15 (6.9)	8 (6.9)	7 (6.9)	0.99
Hematological malignancies	20 (9.2)	7 (6.0)	13 (12.7)	0.09
Bowel perforation	20 (9.2)	9 (7.8)	11 (10.8)	0.44
Acute pancreatitis	5 (2.3)	4 (3.4)	1 (0.9)	0.37
Acute myocardial infarction	2 (0.9)	2 (1.7)	0 (0.0)	0.50
Hepatic cirrhosis	27 (12.4)	14 (12.1)	13 (12.7)	0.88
ARDS in COVID-19 pneumonia	5 (2.3)	1 (0.9)	4 (3.9)	0.19
Others	36 (16.5)	18 (15.5)	18 (17.6)	0.67
Immunosuppression	88 (40.4)	48 (41.4)	40 (39.2)	0.75
Charlson comorbidity index (median; [IQR])	5 (4–7)	5 (4–7)	5 (4–6)	0.62
*Setting* (*n*; [%])				
ICU	74 (33.9)	46 (39.7)	28 (27.5)	0.06
Medical ward	87 (39.9)	47 (40.5)	40 (39.2)	0.84
Hematology	14 (6.4)	4 (3.4)	10 (9.8)	0.09
Surgical ward	43 (19.8)	19 (16.4)	24 (23.5)	0.19
*Pathophysiological conditions*				
Vasopressors (*n*; [%])	38 (17.4)	25 (21.6)	13 (12.7)	0.09
Mechanical ventilation (*n*; [%])	48 (22.0)	31 (26.7)	17 (16.7)	0.07
Baseline CL_CR_ (mL/min/1.73 m^2^; median; [IQR])	68.0 (23.3–101.0)	62.5 (18.0–96.5)	73.0 (30.0–101.0)	0.82
Continuous renal replacement therapy (*n*; [%])	23 (10.6)	16 (13.8)	7 (6.9)	0.10
Intermittent hemodialysis (*n*; [%])	15 (6.9)	9 (7.8)	6 (5.9)	0.59
Augmented renal clearance (*n*; [%])	22 (10.1)	12 (10.3)	10 (9.8)	0.89
*Site of infection* (*n*; [%])				
HAP/VAP	23 (10.6)	16 (13.8)	7 (6.9)	0.10
BSI	100 (45.9)	57 (49.1)	43 (42.2)	0.30
HAP/VAP + BSI	21 (9.6)	11 (9.5)	10 (9.8)	0.94
IAI	17 (7.8)	9 (7.8)	8 (7.8)	0.98
IAI + BSI	15 (6.9)	9 (7.8)	6 (5.9)	0.59
UTI	16 (7.3)	5 (4.3)	11 (10.8)	0.07
UTI + BSI	11 (5.0)	5 (4.3)	6 (5.9)	0.60
SSTI	5 (2.3)	2 (1.7)	3 (2.9)	0.67
BJI	7 (3.2)	1 (0.9)	6 (5.9)	0.06
BJI + BSI	3 (1.4)	1 (0.9)	2 (1.9)	0.60
Overall BSI	150 (68.8)	83 (71.6)	67 (65.7)	0.35
Overall pneumonia	44 (20.2)	27 (23.3)	17 (16.7)	0.23
*Ceftazidime/avibactam MIC* (*n*; [%])				
0.5 mg/L	4 (1.8)	2 (1.7)	2 (1.9)	0.99
1 mg/L	23 (10.6)	10 (8.6)	13 (12.7)	0.32
2 mg/L	93 (42.7)	46 (39.7)	47 (46.2)	0.34
4 mg/L	62 (28.4)	35 (30.2)	27 (26.5)	0.55
8 mg/L	36 (16.5)	23 (19.8)	13 (12.7)	0.16
≥4 mg/L	98 (44.9)	58 (50.0)	40 (39.2)	0.11
*Ceftazidime/avibactam treatment regimens*				
Continuous infusion (*n*; [%])	135 (61.9)	37 (31.9)	98 (96.1)	**<0.001**
Starting daily dosing regimen (mg; median; [IQR])	7,500 (3,750-7,500)	7,500 (3,750-7,500)	7,500 (7,500-7,500)	0.40
Combination therapy (*n*; [%])	94 (43.1)	78 (67.2)	16 (15.7)	**<0.001**
Treatment duration (days; median; [IQR])	12.5 (9.0–15.75)	14 (10–16)	10 (7–15)	**0.01**
*Outcome* (*n*; [%])				
Microbiological eradication^*a*^	121/184 (65.8)	53/100 (53.0)	68/102 (81.0)	**<0.001**
90-Day resistance development	24 (11.0)	18 (15.5)	6 (5.9)	**0.02**
Clinical cure	128 (58.7)	56 (48.3)	72 (70.6)	**<0.001**
30-Day mortality rate	40 (18.3)	26 (22.4)	14 (13.7)	0.10

^*a*^ 184/218 patients had follow-up microbiological cultures, 100/116 and 84/102 in the pre- and post-intervention groups, respectively.

^*b*^ ARDS: acute distress respiratory syndrome; BJI: bone and joint infection; BSI: bloodstream infection; CLCr: creatinine clearance; HAP: hospital-acquired pneumonia; IAI: intraabdominal infection; ICU: intensive care unit; IQR: interquartile range; MIC: minimum inhibitory concentration; SSTI: skin and soft tissue infection; UTI: urinary tract infection; VAP: ventilator-associated pneumonia.

^*c*^ The bold values represents variables having statistical significance.

No significant difference existed in terms of demographics and clinical features between the patients included in the two phases. In the overall population, median (IQR) age was 65.5 (56.3–74.0) years, with a male preponderance (66.5%). The most prevalent underlying disease was solid organ transplantation (57 cases; 26.1%), followed by solid cancer (31 cases; 14.2%) and by hepatic cirrhosis (27 cases; 12.4%). Patients had a median (IQR) CCI of 5 (4–7) and were immunosuppressed in more than one-third of cases (40.4%). Wards of admission were mainly medical (87 cases; 39.9%), followed by intensive care unit (ICU) (74 cases; 33.9%), surgical (43 cases; 19.8%), and hematological (14 cases; 6.4%). CRRT and IHD were applied in 23 (10.6%) and 15 cases (6.9%), respectively, whereas 22 patients (10.1%) had ARC. BSI was the most prevalent type of infection (100 cases; 45.9%), followed by HAP/VAP (23 cases; 10.6%), HAP/VAP plus BSI (21 cases; 9.6%), IAI (17 cases; 7.8%), and UTI (16 cases; 7.3%). All the 218 KPC-Kp isolates were susceptible to ceftazidime/avibactam, with 98 out of them (44.9%) having an MIC of ceftazidime/avibactam ≥ 4 mg/L.

Looking at the ceftazidime/avibactam treatment features being significantly different between the two phases, in the post-intervention phase, the use of CI increased (96.1% vs. 31.9%; *P* < 0.001); that of combination therapy decreased (15.7% vs. 67.2%; *P* < 0.001); and the treatment duration was shortened (median of 10 days vs. 14 days; *P* = 0.01). Specifically, combination therapy was adopted in 78 cases in the pre-intervention phase (45 with meropenem, 15 with tigecycline, six with meropenem + tigecycline, four with colistin + tigecycline, three with tigecycline + gentamycin, and one each with gentamycin, meropenem + fosfomycin, gentamycin + colistin, meropenem + cotrimoxazole, and colistin + cotrimoxazole) and in 16 cases in the post-intervention phase (eight with fosfomycin, six with tigecycline, and one each with gentamycin and meropenem). In the post-intervention phase, 75/102 patients (73.5%) had treatment personalized by means of 145 TDM-guided ECPAs. The median CI ceftazidime/avibactam daily MD was 2 g/0.5 g q8h over 8 h, ranging from 0.5 g/0.125 g q12h over 12 h to 2 g/0.5 g q6h over 6 h. The median (IQR) *f*C_ss_ was 37.8 mg/L (23.5–60.8 mg/L) for ceftazidime and 9.3 mg/L (4.7–15.5 mg/L) for avibactam. Aggressive joint PK/PD target was attained in 61 cases (81.3%) and non-attained in the other 14 (18.7%). No adverse event was reported.

Looking at the outcome features being significantly different between the two phases, the post-intervention phase increased both the microbiological eradication rate (81.0% vs 53.0%; *P* < 0.001) and the clinical cure rate (70.6% vs. 48.3%; *P* < 0.001), whereas the 90-day resistance development decreased (5.9% vs. 15.5%), and the 30-day mortality rate trended to decrease (13.7% vs. 22.4%; *P* = 0.10).

Univariate and multivariate regression analyses testing the potential variables associated with microbiological failure in the pre- and post-intervention phase are reported in Table 2 and 3, respectively. Overall, in the pre-intervention phase, the use of CRRT (odds ratio [OR] 5.20; 95% confidence interval [CI] 1.21–22.34; *P* = 0.027) and the isolation of KPC-Kp strains with a ceftazidime/avibactam MIC value ≥ 4 mg/L (OR 3.08; 95% CI 1.10–8.64; *P* = 0.032) were found to be independent predictors of microbiological failure. Conversely, in the post-intervention phase, aggressive joint PK/PD target attainment (OR 0.03; 95% CI 0.005–0.20; *P* < 0.001) and BSI (OR 0.09; 95% CI 0.02–0.53; *P* = 0.008) were found to be independent predictors of microbiological eradication.

**TABLE 2:** Univariate and multivariate analyses comparing patients having microbiological eradication vs. microbiological failure treated with ceftazidime/avibactam for documented KPC-Kp infections in pre-intervention phase^*b*^^,^^*c*^

Variables	Microbiological eradication (*n* = 53)	Microbiological failure (*n* = 47)	Univariate *P* value	Multivariate analysis^*a*^
*Demographics*				
Age (median; [IQR])	62.0 (54.0–73.0)	64.0 (47.5–69.0)	0.18	
Gender (male/female; *n* [%])	37/16 (69.8/30.2)	33/14 (70.2/29.8)	0.97	
Body mass index (median; [IQR])	24.1 (22.2–27.5)	26.3 (22.2–28.3)	0.33	
Obesity (*n*; [%])	8 (15.1)	9 (19.1)	0.59	
Immunosuppression (*n*; [%])	24 (45.3)	20 (42.6)	0.78	
Charlson Comorbidity Index (Median; [IQR])	6 (4–8)	5 (3–7)	**0.035**	
*Setting* (*n*; [%])				
ICU	11 (11.0)	28 (28.0)	**<0.001**	
No-ICU	42 (42.0)	19 (19.0)		
*Pathophysiological conditions*				
Vasopressors (*n*; [%])	7 (13.2)	15 (31.9)	**0.02**	
Mechanical ventilation (n; [%])	7 (13.2)	21 (44.7)	**<0.001**	
Baseline CL_CR_ (mL/min/1.73 m^2^; median; [IQR])	36.0 (11.0–74.0)	88.5 (56.75–111.5)	**<0.001**	
Continuous renal replacement therapy (*n*; [%])	4 (7.5)	11 (23.4)	**0.047**	**OR 5.20 (95% CI 1.21–22.34**) ***P* = 0.027**
Intermittent hemodialysis (*n*; [%])	7 (13.2)	1 (2.1)	0.06	
Augmented renal clearance (*n*; [%])	2 (3.8)	7 (14.9)	0.08	
*Site of infection* (*n*; [%])				
HAP/VAP	2 (3.8)	9 (19.1)	**0.02**	
BSI	39 (73.7)	17 (36.3)	**<0.001**	
HAP/VAP + BSI	4 (7.5)	7 (14.9)	0.34	
IAI	0 (0.0)	5 (10.6)	**0.02**	
IAI + BSI	4 (7.5)	4 (8.5)	0.99	
UTI	0 (0.0)	2 (4.3)	0.22	
UTI + BSI	4 (7.5)	1 (2.1)	0.37	
SSTI	0 (0.0)	1 (2.1)	0.47	
BJI	0 (0.0)	1 (2.1)	0.47	
*Ceftazidime/avibactam MIC* (*n*; [%])				
≥4 mg/L	20 (37.7)	29 (61.7)	**0.017**	**OR 3.08 (95% CI 1.10–8.64)** ***P* = 0.032**
*Ceftazidime/avibactam treatment regimens*				
Continuous infusion (*n*; [%])	15 (28.3)	18 (38.3)	0.29	
Combination therapy (*n*; [%])	33 (62.3)	38 (80.9)	**0.04**	
Treatment duration (days; median; [IQR])	14 (10–16)	14 (10.5–17.5)	0.87	

^*a*^ Multivariate analysis adjusted for age, gender, and variables with *P* < 0.10 at univariate analysis.

^*b*^ BJI: bone and joint infection; BSI: bloodstream infection; CLCr: creatinine clearance; HAP: hospital-acquired pneumonia; IAI: intraabdominal infection; ICU: intensive care unit; IQR: interquartile range; MIC: minimum inhibitory concentration; SSTI: skin and soft tissue infection; UTI: urinary tract infection; VAP: ventilator-associated pneumonia.

^*c*^ The bold values represents variables having statistical significance.

**TABLE 3:** Univariate and multivariate analyses comparing patients having microbiological eradication vs. microbiological failure treated with ceftazidime/avibactam for documented KPC-Kp infections in post-intervention phase^*c*^^,^^*d*^

Variables	Microbiological eradication (*n* = 68)	Microbiological failure (*n* = 16)	Univariate *P* value	Multivariate analysis^*a*^
*Demographics*				
Age (median; [IQR])	67.0 (60.75–74.0)	63.5 (55.25–75.0)	0.58	
Gender (male/female; *n* [%])	42/26 (61.8/38.2)	11/5 (68.8/31.2)	0.60	
Body mass index (median; [IQR])	25.3 (22.6–29.4)	24.3 (21.6–26.5)	0.20	
Obesity (*n*; [%])	16 (23.5)	2 (12.5)	0.50	
Immunosuppression (*n*; [%])	28 (41.2)	7 (43.8)	0.85	
Charlson comorbidity index (median; [IQR])	5.0 (4.0–6.25)	5.5 (3.75–6.5)	0.92	
*Setting* (*n*; [%])				
ICU	18 (21.4)	5 (6.0)	0.70	
No-ICU	50 (59.5)	11 (13.1)		
*Pathophysiological conditions*				
Vasopressors (*n*; [%])	12 (17.6)	1 (6.3)	0.45	
Mechanical ventilation (*n*; [%])	14 (20.6)	2 (12.5)	0.73	
Baseline CL_CR_ (mL/min/1.73 m^2^; median; [IQR])	70.0 (27.0–96.0)	65.0 (43.5–110.5)	0.57	
Continuous renal replacement therapy (*n*; [%])	7 (10.3)	0 (0.0)	0.34	
Intermittent hemodialysis (*n*; [%])	4 (5.9)	0 (0.0)	0.99	
Augmented renal clearance (*n*; [%])	7 (10.3)	1 (6.3)	0.99	
*Site of infection* (*n*; [%])				
HAP/VAP	2 (2.9)	2 (12.5)	0.16	
BSI	40 (58.8)	3 (18.7)	**0.005**	**OR 0.09 (95% CI 0.02–0.53)** ***P* = 0.008**
HAP/VAP + BSI	7 (10.3)	3 (18.7)	0.39	
IAI	5 (7.4)	1 (6.3)	0.99	
IAI + BSI	4 (5.9)	2 (12.5)	0.32	
UTI	5 (7.4)	2 (12.5)	0.61	
UTI + BSI	4 (5.9)	2 (12.5)	0.32	
SSTI	1 (1.5)	1 (6.3)	0.35	
*Ceftazidime/avibactam MIC* (*n*; [%])				
≥4 mg/L	27 (39.7)	7 (43.8)	0.77	
*Ceftazidime/avibactam treatment regimens*				
Continuous infusion (*n*; [%])	65 (95.6)	16 (100.0)	0.99	
Combination therapy (*n*; [%])	10 (14.7)	2 (12.5)	0.99	
Treatment duration (days; median; [IQR])	10 (7–14)	10.5 (8.0–14.25)	0.47	
Attainment of aggressive PK/PD target (*n*; [%])^*b*^	43/47 (91.5)	6/14 (42.9)	**<0.001**	**OR 0.03 (95% CI 0.005–0.20) *P* < 0.001**

^*a*^ Multivariate analysis adjusted for age, gender, and variables with *P* < 0.10 at univariate analysis.

^*b*^ Overall 61/75 of patients who underwent TDM-guided ECPA program had available follow-up cultures for defining microbiological outcome.

^*c*^ BSI: bloodstream infection; CLCr: creatinine clearance; HAP: hospital-acquired pneumonia; IAI: intraabdominal infection; ICU: intensive care unit; IQR: interquartile range; MIC: minimum inhibitory concentration; PK/PD: pharmacokinetic/pharmacodynamic; SSTI: skin and soft tissue infection; UTI: urinary tract infection; VAP: ventilator-associated pneumonia.

^*d*^ The bold values represents variables having statistical significance.

Univariate and multivariate regression analyses testing the potential variables associated with 90-day resistance development to ceftazidime/avibactam in the pre- and post-intervention phase are summarized in Table 4 and 5, respectively. In the pre-intervention phase, no variable resulted in association at multivariate analysis with resistance development. Noteworthy, in the post-intervention phase, aggressive joint PK/PD target attainment inemerged as independent predictor of reduced risk of 90-day resistance development to ceftazidime/avibactam (OR 0.07; 95% CI 0.01–0.69; *P* = 0.023).

**TABLE 4:** Univariate and multivariate analyses comparing patients having resistance development vs. no resistance development treated with ceftazidime/avibactam for documented KPC-*Klebsiella pneumoniae* infections in pre-intervention phase^*b*^^,^^*c*^

Variables	CAZ-AVI resistance occurrence (*n* = 18)	CAZ-AVI resistance non-occurrence (*n* = 98)	Univariate *P* value	Multivariate analysis^*a*^
*Demographics*				
Age (median; [IQR])	64.5 (38.25–68.25)	64.5 (54.0–74.0)	0.20	
Gender (male/female; *n* [%])	15/3 (83.3/16.7)	67/31 (68.4/31.6)	0.27	
Body mass index (median; [IQR])	25.1 (21.6–28.0)	25.1 (22.4–27.4)	0.90	
Obesity (*n*; [%])	2 (11.1)	16 (16.3)	0.73	
Immunosuppression (*n*; [%])	7 (38.9)	41 (41.8)	0.82	
Charlson comorbidity index (median; [IQR])	4 (1.25–6.5)	5 (4–7)	**0.028**	
*Setting* (*n*; [%])				
ICU	11 (9.5)	35 (35.7)	0.08	
No-ICU	7 (6.0)	63 (64.3)		
*Pathophysiological conditions*				
Vasopressors (*n*; [%])	6 (33.3)	19 (19.4)	0.19	
Mechanical ventilation (*n*; [%])	7 (38.9)	24 (24.5)	0.20	
Baseline CL_CR_ (mL/min/1.73 m^2^; median; [IQR])	100.0 (62.4–125.0)	56.0 (18.0–92.0)	**0.009**	
Continuous renal replacement therapy (*n*; [%])	3 (16.7)	13 (13.3)	0.71	
Intermittent hemodialysis (*n*; [%])	0 (0.0)	9 (9.2)	0.35	
Augmented renal clearance (*n*; [%])	5 (27.8)	7 (7.1)	**0.008**	
*Site of infection* (*n*; [%])				
HAP/VAP	4 (22.2)	12 (12.2)	0.27	
BSI	8 (44.4)	49 (50.0)	0.66	
HAP/VAP + BSI	2 (11.1)	9 (9.2)	0.68	
IAI	1 (5.6)	8 (8.2)	0.99	
IAI + BSI	3 (16.7)	6 (6.1)	0.14	
UTI	0 (0.0)	5 (5.1)	0.99	
UTI + BSI	0 (0.0)	5 (5.1)	0.99	
SSTI	0 (0.0)	2 (2.0)	0.99	
BJI	0 (0.0)	1 (1.0)	0.99	
BJI + BSI	0 (0.0)	1 (1.0)	0.99	
*Ceftazidime/avibactam MIC* (*n*; [%])				
≥4 mg/L	13 (72.2)	45 (45.9)	**0.04**	*OR 4.52 (95% CI 0.93–22.01)* *P = 0.06*
*Ceftazidime/avibactam treatment regimens*				
Continuous infusion (*n*; [%])	11 (61.1)	26 (26.5)	**0.004**	
Combination therapy (*n*; [%])	15 (83.3)	64 (65.3)	0.17	
Treatment duration (days; median; [IQR])	13.0 (10.25–15.75)	14.0 (10.0–15.75)	0.94	

^*a*^ Multivariate analysis adjusted for age, gender, and variables with *P* < 0.10 at univariate analysis.

^*b*^ BJI: bone and joint infection; BSI: bloodstream infection; CLCr: creatinine clearance; HAP: hospital-acquired pneumonia; IAI: intraabdominal infection; ICU: intensive care unit; IQR: interquartile range; MIC: minimum inhibitory concentration; SSTI: skin and soft tissue infection; UTI: urinary tract infection; VAP: ventilator-associated pneumonia.

^*c*^ The bold values represents variables having statistical significance.

**TABLE 5:** Univariate and multivariate analyses comparing patients having resistance development vs. no resistance development treated with ceftazidime/avibactam for documented KPC-*Klebsiella pneumoniae* infections in post-intervention phase^*c*^^,^^*d*^

Variables	CAZ-AVI resistance occurrence (*n* = 6)	CAZ-AVI resistance non-occurrence (*n* = 96)	Univariate *P* value	Multivariate analysis^*a*^
*Demographics*				
Age (median; [IQR])	64.0 (54.75–70.25)	67.0 (60.0–74.0)	0.56	
Gender (male/female; *n* [%])	5/1 (83.3/16.7)	58/38 (60.4/39.6)	0.40	
Body mass index (median; [IQR])	26.1 (21.7–27.6)	25.4 (22.6–29.1)	0.75	
Obesity (*n*; [%])	1 (16.7)	20 (20.8)	0.99	
Immunosuppression (*n*; [%])	3 (50.0)	37 (38.5)	0.68	
Charlson comorbidity index (median; [IQR])	4.5 (3.25–5.0)	5 (4–7)	0.19	
*Setting* (*n*; [%])				
ICU	3 (2.9)	25 (24.5)	0.34	
No-ICU	3 (2.9)	71 (69.7)		
*Pathophysiological conditions*				
Vasopressors (*n*; [%])	1 (16.7)	12 (12.5)	0.57	
Mechanical ventilation (*n*; [%])	2 (33.3)	15 (15.6)	0.26	
Baseline CL_CR_ (mL/min/1.73 m^2^; median; [IQR])	105.5 (80.0–113.0)	70.0 (27.0–99.0)	0.08	
Continuous renal replacement therapy (*n*; [%])	0 (12.5)	7 (7.3)	0.99	
Intermittent hemodialysis (*n*; [%])	0 (0.0)	6 (6.3)	0.99	
Augmented renal clearance (*n*; [%])	1 (16.7)	9 (9.4)	0.47	
*Site of infection* (*n*; [%])				
HAP/VAP	2 (33.3)	5 (5.2)	**0.05**	
BSI	1 (16.7)	42 (43.7)	0.40	
HAP/VAP + BSI	3 (50.0)	7 (7.3)	**0.01**	
IAI	0 (0.0)	8 (8.3)	0.99	
IAI + BSI	0 (0.0)	6 (6.3)	0.99	
UTI	0 (0.0)	11 (11.5)	0.99	
UTI + BSI	0 (0.0)	6 (6.3)	0.99	
SSTI	0 (0.0)	3 (3.1)	0.99	
BJI	0 (0.0)	6 (6.3)	0.99	
BJI + BSI	0 (0.0)	2 (2.0)	0.99	
*Ceftazidime/avibactam MIC* (*n*; [%])				
≥4 mg/L	5 (83.3)	35 (36.5)	**0.03**	
*Ceftazidime/avibactam treatment regimens*				
Continuous infusion (*n*; [%])	6 (100.0)	92 (95.8)	0.99	
Combination therapy (*n*; [%])	1 (16.7)	15 (15.6)	0.99	
Treatment duration (days; median; [IQR])	13.0 (9.0–15.5)	10 (7–15)	0.64	
Attainment of aggressive PK/PD target (*n*; [%])^*b*^	2/6 (33.3)	59/69 (85.5)	**0.01**	**OR 0.07 (95% CI 0.01–0.69)** ***P* = 0.023**

^*a*^ Multivariate analysis adjusted for age, gender, and variables with *P* < 0.10 at univariate analysis.

^*b*^ Overall 75/102 patients underwent TDM-guided ECPA program.

^*c*^ BJI: bone and joint infection; BSI: bloodstream infection; CLCr: creatinine clearance; HAP: hospital-acquired pneumonia; IAI: intraabdominal infection; ICU: intensive care unit; IQR: interquartile range; MIC: minimum inhibitory concentration; PK/PD: pharmacokibetic/pharmacodynamic; SSTI: skin and soft tissue infection; UTI: urinary tract infection; VAP: ventilator-associated pneumonia.

^*d*^ The bold values represents variables having statistical significance.

Univariate and multivariate regression analyses testing the potential variables associated with clinical failure in the pre- and post-intervention phase are reported in Table S1 and S2, respectively. Overall, in the pre-intervention phase, ICU admission (OR 3.55; 95% CI 1.18–10.69; *P* = 0.024) and ARC occurrence (OR 8.34; 95% CI 1.00–69.27; *P* = 0.049) were found to be independent predictors of clinical failure. In the post-intervention phase, aggressive joint PK/PD target attainment was found to be an independent predictor of clinical cure (OR 0.08; 95% CI 0.02–0.34; *P* < 0.001), whereas HAP/VAP was associated with an increased risk of clinical failure (OR 11.61; 95% CI 1.55–87.06; *P* = 0.017).

## DISCUSSION

To the best of our knowledge, this is the first study that investigated, by means of a pre-post quasi-experimental study, the impact of a multidisciplinary intervention of antimicrobial stewardship aimed at attaining an aggressive joint PK/PD target with ceftazidime/avibactam on treatment outcome of documented KPC-Kp infections and on prevention of ceftazidime/avibactam resistance development.

Overall, the patient populations included in the two phases were quite similar in terms of demographics and clinical features. The comparative findings may support the contention that a multidisciplinary intervention of antimicrobial stewardship aiming at an aggressive joint PK/PD target attainment based on delivering ceftazidime/avibactam by CI and adopting a real-time TDM-guided strategy may be valuable either for improving the clinical/microbiological outcome of KPC-Kp infections treated with ceftazidime/avibactam or for reducing the prevalence rate of 90-day resistance development to ceftazidime/avibactam.

In the pre-intervention phase, ceftazidime/avibactam was mainly delivered by II used in combination therapy, and the median treatment duration was 14 days. Multivariate analysis found that applying CRRT and having infections sustained by KPC-Kp strains with borderline susceptibility to ceftazidime/avibactam (namely, an MIC ≥ 4 mg/L) were the two variables being independently associated with an increased risk of microbiological failure. ICU admission and ARC occurrence were associated with an increased risk of clinical failure. It may be speculated that during this phase, high rates of aggressive joint PK/PD target non-attainment could have occurred due to the prevalent use of standard dosing regimens of ceftazidime/avibactam delivered by II. This could have represented the common underlying mechanism shared by the aforementioned conditions in favoring microbiological and clinical failure and in trending to favor resistance development to ceftazidime/avibactam. In agreement with this hypothesis, a recent meta-analysis testing variables potentially impacting the risk of aggressive PK/PD target non-attainment of beta-lactams in critically ill patients showed that this risk was increased by the presence of gram-negative isolates with borderline susceptibility to beta-lactams and decreased by the use of beta-lactams in prolonged/CI (9). Additionally, a retrospective study involving 77 patients being treated with ceftazidime/avibactam for CRE infections found that CRRT was an independent predictor of microbiological failure and resistance development (OR 26.67; 95% CI 2.24–317.1; *P* = 0.009) (24).

In the post-intervention phase, the steps of the antimicrobial stewardship intervention agreed upon by the multidisciplinary team were applied in the majority of cases. Ceftazidime/avibactam was mainly delivered by CI used in monotherapy with a posology optimized by means of TDM-guided ECPAs, and median treatment duration was shortened to 10 days. Importantly, the aggressive joint PK/PD target was attained in the vast majority of cases and revealed as the only variable associated with all of the three positive outcomes, namely, microbiological eradication, protection against 90-day resistance development to ceftazidime/avibactam, and clinical cure. BSI was associated with microbiological eradication and HAP/VAP with clinical failure risk.

Since its introduction in the therapeutic armamentarium, the use of ceftazidime/avibactam at the standard licensed dosages was shown to be potentially associated with an increased risk of resistance development. In this regard, an early Greek study explored the epidemiology of BSIs by carbapenemase-producing *K. pneumoniae* among ICU patients after the introduction of ceftazidime/avibactam in January 2018 (29). Multivariate analysis showed that a prior administration of ceftazidime/avibactam independently predicted developing BSI caused by ceftazidime/avibactam-resistant isolates (OR 16.7, 95% CI 1.8–158.6; *P* = 0.014) (29). Unfortunately, this latter may be a quite common scenario in complex settings like ours in which the prevalence of ceftazidime/avibactam resistance may nowadays worryingly achieve rates as high as 15–20% (5–7). Even in our study, the prevalence of 90-day resistance to ceftazidime/avibactam in the pre-intervention phase had a similar magnitude rate (15.5%). Noteworthy, it significantly decreased (*P* = 0.02) by around threefold in the post-intervention phase (5.9%). This suggests that our intervention focused on aggressive joint PK/PD target attainment may represent an effective tool for counteracting this tendency, potentially becoming a mandatory issue for extending the lifetime of ceftazidime/avibactam. This is in agreement with a recent meta-analysis showing that attaining aggressive vs. conservative PK/PD targets may be highly protective against the risk of resistance development to beta-lactams (OR 0.06; 95% CI 0.01–0.29) when treating gram-negative infections with beta-lactams in critically ill patients (9).

Several studies showed that CI may improve the likelihood of aggressive PK/PD target attainment with beta-lactams compared to delivering the same daily dose intermittently and may favor better outcomes (12, 14, 30). Among patients having KPC-Kp infections being treated with ceftazidime/avibactam, the use of prolonged infusion was shown to be an independent predictor of reduced mortality risk (OR 0.54; 95% CI 0.34–0.83; *P* = 0.006) (12). Unfortunately, delivering ceftazidime/avibactam by CI could not by itself warrant always aggressive joint PK/PD target attainment. A recent innovative population PK/PD study aimed at attaining aggressive joint PK/PD target of ceftazidime/avibactam against KPC- and OXA-48-producing *Enterobacterales* showed that adjusting CI dosing regimen in critically ill patients based solely on estimated CL_CR_ might be suboptimal (15). This is due to the fact that the likelihood of aggressive joint PK/PD target attainment may be hindered by a much faster avibactam than ceftazidime elimination, namely a condition experienced especially by patients without renal dysfunction (16). Conversely, the use of higher daily doses delivered by CI and adjusted by means of TDM-guided ECPAs may have the potential to maximize the likelihood of aggressive joint PK/PD target attainment against KPC-Kp infections (15). In agreement with this, in the post-intervention phase, aggressive joint PK/PD targets were attained in the vast majority of patients undergoing TDM-guided ECPA. Prevalent reasons for non-attainment were the borderline susceptibility of clinical isolates in some cases and the presence of ARC in others.

Finally, the findings may support once more the contention that antimicrobial stewardship interventions aimed at maximizing PK/PD target attainment of ceftazidime/avibactam may represent a valuable approach for avoiding the need for using combination therapy and for shortening treatment duration in KPC-Kp infections, as just previously suggested (12, 13, 31–33).

Limitations of our study should be recognized. The retrospective monocentric study design could limit the generalizability of our findings to other settings. The unavailability of microbiological follow-up cultures in approximately 15% of cases could represent a selection bias. Compliance with the multidisciplinary approach was high but unfortunately incomplete. Only total ceftazidime and avibactam concentrations were measured, and the free fractions were only calculated by considering the plasma protein binding retrieved in the literature. Conversely, the large sample size in both phases, the similarity in terms of demographics and clinical features between patients included in the two phases, and the use of an adjusted multivariate regression model may represent points of strength.

In conclusion, our findings suggest that implementing a multidisciplinary intervention aimed at attaining aggressive joint PK/PD target by using ceftazidime/avibactam in CI coupled with TDM-guided ECPAs could represent an effective strategy for improving clinical/microbiological outcome in treating KPC-Kp infections with monotherapy and could significantly reduce the prevalence of resistance development to ceftazidime/avibactam. Prospective confirmatory studies are warranted.

## SUPPLEMENTAL MATERIAL

Supplemental material aac.00488-25-s0001.docx Table S1 and S2.

ASM does not own the copyrights to Supplemental Material that may be linked to, or accessed through, an article. The authors have granted ASM a non-exclusive, world-wide license to publish the Supplemental Material files. Please contact the corresponding author directly for reuse.
