# Supplementary material for: A pre-post quasi-experimental study of antimicrobial stewardship exploring the impact of a multidisciplinary approach aimed at attaining an aggressive joint pharmacokinetic/pharmacodynamic target with ceftazidime/avibactam on treatment outcome of KPC-producing Klebsiella pneumoniae infections and on ceftazidime/avibactam resistance development
Source: Antimicrob Agents Chemother. 2025 Jun 6;69(7):e00488-25. doi: 10.1128/aac.00488-25 (PMC12217479; doi:10.1128/aac.00488-25)
Supplement: Supplemental material — Table S1 and S2. [file aac.00488-25-s0001.docx]

**Supplementary materials**

**Supplementary Table 1 –** Univariate and multivariate analysis comparing patients having clinical cure vs. clinical failure treated with ceftazidime/avibactam for documented KPC-Kp infections in pre-intervention phase

**Supplementary Table 2 –** Univariate and multivariate analysis comparing patients having clinical cure vs. clinical failure treated with ceftazidime/avibactam for documented KPC-Kp infections in post-intervention phase

| **Supplementary Table 1 –** Univariate and multivariate analysis comparing patients having clinical cure vs. clinical failure treated with ceftazidime/avibactam for documented KPC-Kp infections in pre-intervention phase | | | | |
| --- | --- | --- | --- | --- |
| **Variables** | **Clinical cure**  **(n=56)** | **Clinical failure**  **(n=60)** | **Univariate p value** | **Multivariate analysis*** |
| *Demographics* | | | | |
| Age (Median; [IQR]) | 64.0 (53.0-73.0) | 65.0 (53.75-74.5) | 0.99 |  |
| Gender (male/female; n [%]) | 39/17 (69.6/30.4) | 43/17 (71.7/28.3) | 0.81 |  |
| Body mass index (Median; [IQR]) | 24.1 (22.0-26.9) | 25.8 (22.6-28.2) | 0.19 |  |
| Obesity (n; [%]) | 8 (14.3) | 10 (19.1) | 0.72 |  |
| Immunosuppression (n; [%]) | 24 (42.9) | 24 (40.0) | 0.76 |  |
| Charlson Comorbidity Index (Median; [IQR]) | 6 (4-7.25) | 5 (3-7) | 0.20 |  |
| *Setting* (n; [%]) | | | | |
| ICU | 10 (8.6) | 36 (31.0) | **<0.001** | **OR 3.55 (95%CI 1.18-10.69)**  **p=0.024** |
| No-ICU | 46 (39.7) | 24 (20.7) |  |  |
| *Pathophysiological conditions* | | | | |
| Vasopressors (n; [%]) | 6 (10.7) | 19 (31.7) | **0.006** |  |
| Mechanical ventilation (n; [%]) | 7 (12.5) | 24 (40.0) | **<0.001** |  |
| Baseline CL_CR_ (mL/min/1.73m^2^; Median; [IQR]) | 50.0 (15.0-84.0) | 75.0 (28.0-113.0) | **0.018** |  |
| Continuous renal replacement therapy (n; [%]) | 3 (5.4) | 13 (21.7) | **0.01** |  |
| Intermittent hemodialysis (n; [%]) | 7 (12.5) | 2 (3.3) | 0.09 |  |
| Augmented renal clearance (n; [%]) | 2 (3.6) | 10 (16.7) | **0.03** | **OR 8.34 (95%CI 1.00-69.27)**  **p=0.049** |
| *Site of infection* (n; [%]) | | | | |
| HAP/VAP | 3 (5.4) | 13 (21.7) | **0.01** |  |
| BSI | 34 (60.7) | 23 (38.3) | **0.016** |  |
| HAP/VAP + BSI | 2 (3.6) | 9 (15.0) | **0.05** |  |
| IAI | 5 (8.9) | 4 (6.7) | 0.74 |  |
| IAI + BSI | 5 (8.9) | 4 (6.7) | 0.74 |  |
| UTI | 2 (3.6) | 3 (5.0) | 0.99 |  |
| UTI + BSI | 4 (7.1) | 1 (1.7) | 0.20 |  |
| SSTI | 1 (1.8) | 1 (1.7) | 0.99 |  |
| BJI | 0 (0.0) | 1 (1.7) | 0.99 |  |
| *Ceftazidime/avibactam MIC* (n; [%]) | | | | |
| ≥4 mg/L | 24 (42.9) | 34 (56.7) | 0.14 |  |
| *Ceftazidime/avibactam treatment regimens* | | | | |
| Continuous infusion (n; [%]) | 14 (25.0) | 23 (38.3) | 0.13 |  |
| Combination therapy (n; [%]) | 32 (57.1) | 46 (76.7) | **0.026** |  |
| Treatment duration (days; Median; [IQR]) | 14 (10-15) | 14 (10-18) | 0.43 |  |
| BJI: bone and joint infection; BSI: bloodstream infection; CI: continuous infusion; CLCr: creatinine clearance; HAP: hospital-acquired pneumonia; IAI: intraabdominal infection; ICU: intensive care unit; IQR: interquartile range; MIC: minimum inhibitory concentration; SSTI: skin and soft tissue infection; UTI: urinary tract infection; VAP: ventilator-associated pneumonia  *Multivariate analysis adjusted for age, gender, and variables with p<0.10 at univariate analysis | | | | |

| **Supplementary Table 2 –** Univariate and multivariate analysis comparing patients having clinical cure vs. clinical failure treated with ceftazidime/avibactam for documented KPC-Kp infections in post-intervention phase | | | | |
| --- | --- | --- | --- | --- |
| **Variables** | **Clinical cure**  **(n=72)** | **Clinical failure**  **(n=30)** | **Univariate p value** | **Multivariate analysis*** |
| *Demographics* | | | | |
| Age (Median; [IQR]) | 66.0 (60.0-72.25) | 68.5 (57.75-74.75) | 0.50 |  |
| Gender (male/female; n [%]) | 45/27 (62.5/37.5) | 18/12 (60.0/40.0) | 0.81 |  |
| Body mass index (Median; [IQR]) | 25.4 (22.8-29.2) | 25.2 (21.9-27.8) | 0.57 |  |
| Obesity (n; [%]) | 15 (20.8) | 6 (20.0) | 0.92 |  |
| Immunosuppression (n; [%]) | 30 (41.7) | 10 (33.3) | 0.43 |  |
| Charlson Comorbidity Index (Median; [IQR]) | 5 (4-6) | 5.5 (4-7) | 0.42 |  |
| *Setting* (n; [%]) | | | | |
| ICU | 15 (14.7) | 13 (12.7) | **0.02** |  |
| No-ICU | 57 (55.9) | 17 (16.7) |  |  |
| *Pathophysiological conditions* | | | | |
| Vasopressors (n; [%]) | 6 (8.3) | 7 (23.3) | **0.039** |  |
| Mechanical ventilation (n; [%]) | 10 (13.9) | 7 (23.3) | 0.25 |  |
| Baseline CL_CR_ (mL/min/1.73m^2^; Median; [IQR]) | 75.5 (33.0-102.0) | 64.0 (24.0-96.0) | 0.27 |  |
| Continuous renal replacement therapy (n; [%]) | 4 (5.6) | 3 (10.0) | 0.42 |  |
| Intermittent hemodialysis (n; [%]) | 5 (6.9) | 1 (3.3) | 0.67 |  |
| Augmented renal clearance (n; [%]) | 9 (8.8) | 1 (3.3) | 0.27 |  |
| *Site of infection* (n; [%]) | | | | |
| HAP/VAP | 2 (2.8) | 5 (16.7) | **0.02** | **OR 11.61 (95%CI 1.55-87.06)**  **p=0.017** |
| BSI | 36 (50.0) | 7 (23.3) | **0.01** |  |
| HAP/VAP + BSI | 4 (5.6) | 6 (20.0) | 0.06 |  |
| IAI | 6 (8.3) | 2 (6.7) | 0.99 |  |
| IAI + BSI | 4 (5.6) | 2 (6.7) | 0.99 |  |
| UTI | 7 (9.7) | 4 (13.3) | 0.73 |  |
| UTI + BSI | 4 (5.6) | 2 (6.7) | 0.99 |  |
| SSTI | 1 (1.5) | 1 (6.3) | 0.50 |  |
| BJI | 5 (6.9) | 1 (6.3) | 0.67 |  |
| BJI + BSI | 2 (2.8) | 0 (0.0) | 0.99 |  |
| *Ceftazidime/avibactam MIC* (n; [%]) | | | | |
| ≥4 mg/L | 27 (37.5) | 13 (43.3) | 0.58 |  |
| *Ceftazidime/avibactam treatment regimens* | | | | |
| Continuous infusion (n; [%]) | 70 (97.2) | 28 (93.3) | 0.58 |  |
| Combination therapy (n; [%]) | 12 (16.7) | 4 (13.3) | 0.77 |  |
| Treatment duration (days; Median; [IQR]) | 10.5 (7.75-19.5) | 9.5 (7.0-13.75) | 0.08 |  |
| Attainment of aggressive PK/PD target** (n; [%]) | 45/50 (90.0) | 16/25 (64.0) | **0.01** | **OR 0.08 (95%CI 0.02-0.34)**  **p<0.001** |
| BJI: bone and joint infection; BSI: bloodstream infection; CI: continuous infusion; CLCr: creatinine clearance; HAP: hospital-acquired pneumonia; IAI: intraabdominal infection; ICU: intensive care unit; IQR: interquartile range; MIC: minimum inhibitory concentration; PK/PD: pharmacokinetic/pharmacodynamic; SSTI: skin and soft tissue infection; UTI: urinary tract infection; VAP: ventilator-associated pneumonia  *Multivariate analysis adjusted for age, gender, and variables with p<0.10 at univariate analysis  ** Overall 75/102 of patients underwent TDM-guided ECPA program | | | | |
